# Changing patterns of cancer incidence in the early- and late-HAART periods: the Swiss HIV Cohort Study

**DOI:** 10.1038/sj.bjc.6605756

**Published:** 2010-06-29

**Authors:** S Franceschi, M Lise, G M Clifford, M Rickenbach, F Levi, M Maspoli, C Bouchardy, S Dehler, G Jundt, S Ess, A Bordoni, I Konzelmann, H Frick, L Dal Maso, L Elzi, H Furrer, A Calmy, M Cavassini, B Ledergerber, O Keiser, the Swiss HIV Cohort Study

**Affiliations:** 1 International Agency for Research on Cancer, 150 cours Albert Thomas, 69372 Lyon cedex 08, France; 2 Epidemiology and Biostatistics Unit Aviano Cancer Center, Via Franco Gallini 2, 33081 Aviano, Italy; 3 Coordination and Data Center, Swiss HIV Cohort Study, Mont-Paisible 16, CHUV, 1011 Lausanne, Switzerland; 4 Cancer Registry of the Canton of Vaud, CHUV Falaises 1, 1011 Lausanne, Switzerland; 5 Cancer Registry of the Canton of Neuchatel, Ave des Cadolles, 2000 Neuchatel, Switzerland; 6 Cancer Registry of the Canton of Geneva, Bd de la Cluse 55, 1205 Geneva, Switzerland; 7 Cancer Registry of the Canton of Zurich, Vogelsangstr. 10, 8091 Zurich, Switzerland; 8 Cancer Registry of Basel, Schönbeinstr. 40, 4003 Basel, Switzerland; 9 Cancer Registry of St Gallen and Appenzell, Flurhofstr. 7, 9000 St Gallen, Switzerland; 10 Cancer Registry of the Canton of Ticino, 6604 Locarno, Switzerland; 11 Cancer Registry of the Canton of Valais, Av Grand Champsec 86, 1950 Sion, Switzerland; 12 Cancer Registry of the Canton of Graubunden, Lostr. 170, 7000 Chur, Switzerland; 13 Division of Infectious Diseases and Hospital Epidemiology, University Hospital Basel, Petersgraben 4, 4031 Basel, Switzerland; 14 Division of Infectious Diseases, University Hospital, University of Bern, Inselspital PKT2B, 3010, Bern, Switzerland; 15 Division of Infectious Disease/HIV Unit, University Hospital Geneva, Rue Micheli-Du-Crest 24, 1205 Geneva, Switzerland; 16 Division of Infectious Diseases, University Hospital Lausanne, Rue du Bugnon 4, 1011 Lausanne, Switzerland; 17 Division of Infectious Diseases and Hospital Epidemiology, University Hospital Zurich, University of Zurich, Rämistrasse 100, 8091 Zurich, Switzerland; 18 Institute of Social and Preventive Medicine, University of Bern, Finkenhubelweg 1, 3012 Bern, Switzerland

**Keywords:** HIV, HAART, age, incidence rates, Switzerland

## Abstract

**Background:**

The advent of highly active antiretroviral therapy (HAART) in 1996 led to a decrease in the incidence of Kaposi's sarcoma (KS) and non-Hodgkin's lymphoma (NHL), but not of other cancers, among people with HIV or AIDS (PWHA). It also led to marked increases in their life expectancy.

**Methods:**

We conducted a record-linkage study between the Swiss HIV Cohort Study and nine Swiss cantonal cancer registries. In total, 9429 PWHA provided 20 615, 17 690, and 15 410 person-years in the pre-, early-, and late-HAART periods, respectively. Standardised incidence ratios in PWHA *vs* the general population, as well as age-standardised, and age-specific incidence rates were computed for different periods.

**Results:**

Incidence of KS and NHL decreased by several fold between the pre- and early-HAART periods, and additionally declined from the early- to the late-HAART period. Incidence of cancers of the anus, liver, non-melanomatous skin, and Hodgkin's lymphoma increased in the early- compared with the pre-HAART period, but not during the late-HAART period. The incidence of all non-AIDS-defining cancers (NADCs) combined was similar in all periods, and approximately double that in the general population.

**Conclusions:**

Increases in the incidence of selected NADCs after the introduction of HAART were largely accounted for by the ageing of PWHA.

The introduction of highly active antiretroviral therapy (HAART) in 1996 has greatly influenced the pattern of cancer incidence in people with HIV or AIDS (PWHA). The decrease in the prevalence of severe immunodeficiency has resulted in a rapid reduction in Kaposi's sarcoma (KS) (Ledergerber *et al*, 1999; Franceschi *et al*, 2008; Polesel *et al*, 2008) and non-Hodgkin's lymphoma (NHL) incidence (Polesel *et al*, 2008; The COHERE Study Group, 2009). However, improved PWHA survival has been accompanied by an increase in the burden of non-AIDS-defining cancers (NADCs) (Engels *et al*, 2008).

The Swiss HIV Cohort Study (SHCS) is a large prospective cohort that has been ongoing for 20 years and is characterised by detailed follow-up information, excellent treatment standards, and the possibility to link with population-based cancer registries. As such, it offers an especially good opportunity to monitor changes in the pattern of cancer incidence in PWHA (Clifford *et al*, 2005; Franceschi *et al*, 2008; Polesel *et al*, 2008). It is now possible to add four additional years of follow-up to our linkage study of the SHCS and Swiss cancer registries, and thus to evaluate the long-term consequences of HAART use, particularly with respect to NADCs.

The aim of our present report is to evaluate the changes in patterns of cancer incidence in the SHCS in three different periods (pre-HAART, 1985–1996; early HAART, 1997–2001; and late HAART: 2002–2006), while taking into account large shifts in the age distribution of PWHA.

## Materials and methods

The SHCS has been enrolling PWHA who are >16 years of age from seven large hospitals in different Swiss cities (Basel, Bern, Geneva, Lausanne, Lugano, St Gallen, and Zurich) since 1988, with some retrospective enrolment going back to 1985 (The Swiss HIV Cohort Study, 2010). Once written informed consent is obtained, detailed information (including demographic and clinical characteristics) is collected. Follow-up visits are scheduled at 6-month intervals and include updating information on selected disease diagnoses, laboratory test results, and HIV/AIDS-related treatments. Only AIDS-defining cancers (KS, NHL, and, since 1993, invasive cervical carcinoma) (Ancelle-Park, 1993) and Hodgkin's lymphoma have been systematically reported in the SHCS since the beginning of the study.

Nine active cancer registries, covering 56% of the Swiss population, collect population-based quality-checked data on cancer incidence in Switzerland (Curado *et al*, 2007). The Cancer Registries of the Cantons of Basel, Geneva, St Gallen and Appenzell, Ticino, Vaud, and Zurich overlap directly with six of the seven regions covered by SHCS centres (all except the Bern SHCS Centre) (Clifford *et al*, 2005).

Swiss cancer registries vary greatly both in size of population coverage (from 165 000 in Neuchatel to 1168 000 in Zurich) and year of initiation of cancer registration (from 1970 in Geneva to 1996 in Ticino). Routine indicators of data completeness and quality in the Swiss registries are very good (Parkin *et al*, 2002; Curado *et al*, 2007).

Record linkage was performed using an upgraded version of an *ad hoc* software application that was previously designed and validated in Italy and Switzerland (Geneva) to match individuals from AIDS and cancer registries while protecting patient anonymity (Dal Maso *et al*, 2001; Clifford *et al*, 2005). Each SHCS centre holds its own nominal participant records. Thus, each SHCS database was independently matched with the corresponding cantonal cancer registry. Furthermore, to account for inter-cantonal migration and health-care mobility, cross-cantonal linkage within neighbouring cancer registries, including those that do not overlap with SHCS centres (i.e., Neuchatel, Valais, and Glarus Graubunden), was also conducted. Personal identifiers were never made visible during linkage procedures, nor were they included in the output file.

From January 1985 through May 2007, 14 560 PWHA (median age, 33 years; 95% range, 22–54) were enrolled in the SHCS. PWHA were excluded (in hierarchical order) from this report if they: (1) did not report a legal residence in a canton covered by a cancer registry with which their SHCS records were linked (*n*=4512, principally those from the Bern SHCS Centre); (2) were followed by a private physician and were unavailable for name-based linkage (*n*=60); (3) their follow-up in the SHCS did not correspond to years for which data were available in cancer registries (*n*=520); or (4) were <16 years (*n*=4) or >69 years of age (*n*=35) at enrolment. Eventually, 9429 PWHA (median age, 32 years; 95% range, 22–53) were included. Of these, 29% were women and 18.1% had AIDS at study entry. For each PWHA included in the study, the relevant time period for the calculation of person-years at risk began 3 months after the date of SHCS enrolment and ended at 70 years of age, the date of last SHCS information, cancer diagnosis, or death, whichever was earliest.

Observed cancers included incident cases reported to cancer registries during the above-defined person-years at risk. In all, 103 cases of KS, 23 NHL, 3 invasive cervical carcinomas, and 10 NADCs diagnosed between 0 and 3 months after SHCS enrolment were considered prevalent cases and were not included. Cancers were classified according to the *International Classification of Diseases for Oncology*, *Third Edition* (Fritz *et al*, 2000), and according to the *International Classification of Diseases and Related Health Problems*, *10th Revision* (World Health Organisation, 1992).

This study was approved by the ethics committees of the SHCS and the International Agency for Research on Cancer. All patients in the SHCS gave written informed consent.

### Statistical methods

Expected numbers of cancers were computed from cancer registry-, sex-, age-, and period-specific incidence rates (IRs) (Parkin *et al*, 1992, 1997, 2002; Curado *et al*, 2007) for three periods (pre-HAART, 1985–1996; early HAART, 1997–2001; late HAART, 2002–2006). Observed numbers of cancers in PWHA were compared with expected numbers by standardised incidence ratios (SIRs). Corresponding 95% confidence intervals (CIs) were computed using the Poisson distribution (Breslow and Day, 1987). All cancers other than KS, NHL, and invasive cervical carcinoma were grouped as NADCs. AIDS-defining cancers and NADCs were further combined into an ‘all cancers’ category.

The IRs among PWHA were also computed separately for the pre-, early-, and late-HAART periods. As the purpose was to make comparisons within the SHCS, IRs were standardised for age (5-year groups) and sex (when appropriate), using the direct method (Breslow and Day, 1987) and the age and sex structure of all PWHA included in this study as a standard population. The 95% CIs of IRs and of IR ratios in different periods were computed according to the Poisson distribution.

Age-specific IRs (i.e., 25–34, 35–44 and 45–54, and 55–69 years) and corresponding 95% CIs were computed for selected NADCs separately in two periods (the pre-HAART and the HAART periods). Age-specific IRs were internally standardised for age (5-year groups) and sex (when appropriate) based on the distribution of all PWHA included in this study. For comparison purposes, the corresponding age-specific IRs published for 1998–2002 in the general population from the same Swiss cantons were also computed (Curado *et al*, 2007).

## Results

Among 9429 SHCS participants followed for a total of 53 715 person-years, 858 cancers were identified (Table 1). Observed and expected numbers and corresponding SIRs in the pre-, early-, and late-HAART periods are shown for cancer sites or types with at least two cases observed. The SIRs of KS, NHL, and invasive cervical carcinoma declined by several fold. The SIRs for some individual NADCs (i.e., anus, liver, and non-melanomatous skin) increased between the pre- and early-HAART periods, but not between the early- and late-HAART periods. The SIR for Hodgkin's lymphoma increased greatly between the pre- (9.2) and the early-HAART (21.0) periods, but little thereafter (28.1). The SIRs for cancer of the head and neck and the lung did not change, whereas the SIR for multiple myeloma declined. The SIRs for all NADCs combined did not substantially change by period. The SIR for the combination of all cancers diminished from 17.6 (95% CI, 16.1–19.1) in the pre-HAART to 3.0 (95% CI, 2.6–3.6) in the late-HAART period (Table 1).

Table 2 is restricted to cancers with >10 cases observed after the introduction of HAART. Age-standardised IRs in the pre-, early-, and late-HAART periods with corresponding IR ratios are shown. Large decreases of IRs for KS, NHL, and all cancers were observed between the pre- and early-HAART periods, whereas no significant change was found for NADCs (IR ratio, 1.2; 95% CI, 0.9–1.7). Increases were observed, however, from the pre- to early-HAART period, for cancer of the anus (IR ratio, 5.2; 95% CI, 1.3–21.3) and non-melanomatous skin cancer (IR ratio, 2.4; 95% CI, 1.0–5.7). When IRs of selected cancers in the early- and late-HAART periods were compared, additional declines in KS (IR ratio, 0.3; 95% CI, 0.2–0.7), NHL (IR ratio, 0.4; 95% CI, 0.2–0.6) and all cancers (IR ratio, 0.6; 95% CI, 0.4–0.8) were found. The IR ratios (late- *vs* early-HAART period) for individual NADCs or their combinations were close to unity (lung, non-melanomatous skin, Hodgkin's lymphoma) or non-significantly below unity (head and neck, anus and liver) (Table 2).

**Figure 1:**
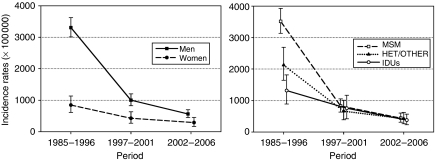
Incidence rates (standardised by 5-year age group and, when appropriate, sex, based on the age and sex distribution of all people with HIV or AIDS (PWHA) included in this study) and corresponding 95% confidence intervals of all cancers among PWHA by sex or HIV-transmission category and period of cancer diagnosis. MSM, men having sex with men; HET, heterosexual; IDU, intravenous drug user.

Figure 1 shows the steep downward trends in the IRs for all cancers, by gender. Decreases were greater among men than women and among men having sex with men than heterosexuals and intravenous drug users. The IRs for all cancers in the two sexes and in the three HIV-transmission categories became similar in the late-HAART period (Figure 1).

**Figure 2:**
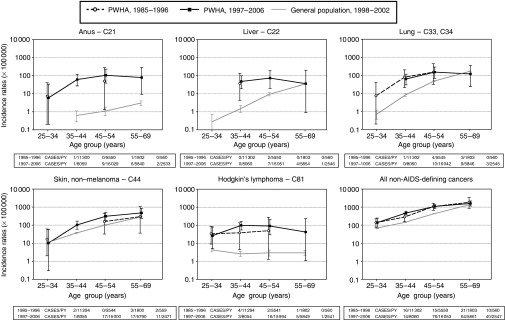
Age-specific incidence rates of selected cancers among people with HIV or AIDS (PWHA) and the general population from the same Swiss Cantons (age-standardised as in Figure 1) by period of cancer diagnosis. PY, person-years.

Age-specific IRs for cancer of the anus, liver, lung, non-melanomatous skin, Hodgkin's lymphoma, and all NADCs combined in PWHA in the pre-HAART and the HAART periods, and, for comparison purposes, in the general population (1998–2002), are shown in Figure 2. Cancer cases were extremely few in the pre-HAART period, especially in PWHA aged ⩾35 years. The 95% CIs of age-specific IRs for individual NADCs in PWHA in the pre-HAART and HAART periods always overlapped. In the HAART periods, 95% CIs of IRs for cancer of the anus, liver, lung, non-melanomatous skin, Hodgkin's lymphoma, and all NADCs combined did not overlap with IRs from the general population in the 35–44- and 45–54-year age groups. In the 55-69-year age group, only for anal cancer did the IR in the HAART periods not overlap with the corresponding IR in the general population (Figure 2).

## Discussion

In this paper, we used SIRs to compare cancer excess in PWHA with that in previous studies (Clifford *et al*, 2005; van Leeuwen *et al*, 2009; Dal Maso *et al*, 2009), and IRs to evaluate cancer trends within the SHCS. Trends in cancer incidence in SHCS participants in the last two decades has been dominated by the favourable trends in KS and NHL. All cancer incidence (including both AIDS-defining cancers and NADCs) decreased in the late-HAART period by 81% compared with the pre-HAART period. Furthermore, mainly because of the decline in KS, previously large differences in cancer incidence between men and women, as well as between men having sex with men, heterosexuals, and intravenous drug users, have disappeared.

Further decreases in the incidence of KS and NHL also emerged between the early- and late-HAART periods. In the late-HAART period, treatment options increased and the risk of untreated viraemia became better appreciated than before (Hammer *et al*, 2008). Virological and immunological outcomes of initial HAART improved between 2000–2001 and 2004–2005 in the SHCS (Vo *et al*, 2008). Indeed, a higher proportion of KS and NHL occurred in HAART-naïve PWHA in the early-HAART (46%) than in the late-HAART (27%) period (data not shown).

This study also showed that the incidence of all NADCs combined did not increase between the pre-HAART and HAART periods, even though by the late-HAART period it had become higher than the declining incidence of KS and NHL combined. In other studies using differently defined populations (e.g., HIV-positive people or people with AIDS only) and different statistical approaches (SIR or age-standardised IRs), increases in NADCs have been reported since the introduction of HAART (Engels *et al*, 2008; Patel *et al*, 2008; Dal Maso *et al*, 2009; Guiguet *et al*, 2009; van Leeuwen *et al*, 2009), especially for cancer of the anus (Piketty *et al*, 2008; Chaturvedi *et al*, 2009), liver (Rosenthal *et al*, 2007; Polesel *et al*, 2010), lung (Bower *et al*, 2003; Kirk *et al*, 2007), and Hodgkin's lymphoma (Biggar *et al*, 2006). Thus, concerns have been raised that HAART or its consequences (immune reconstitution) may have directly contributed to the increase of certain NADCs (Hammer *et al*, 2008).

Drugs included in HAART have been attributed to both carcinogenic (e.g., incorporation of zidovudine into nuclear DNA and alteration of the expression of several cell-cycle genes) (Olivero, 2007) and anti-tumour (anti-angiogenic effect of protease inhibitors) (Monini *et al*, 2004) effects. Zidovudine and zalcitabine have been classified in an International Agency for Research on Cancer Monograph (IARC, 2000) as ‘possibly carcinogenic to humans’ on the basis of inadequate evidence in humans, but sufficient evidence of carcinogenicity in experimental animals (IARC, 2000). The hepatotoxicity of HAART was suspected to worsen the effect of hepatitis B and C infection on the risk of liver cancer (Puoti *et al*, 2004). Hodgkin's lymphoma was reported to occur more frequently in AIDS patients with moderate than severe immunosuppression in the United States (Biggar *et al*, 2006), as well as among those using non-nucleoside reverse transcription inhibitors in the United Kingdom (Powles *et al*, 2009). The possibility of some adverse effect of HAART on cancer risk will be raised again, and it is therefore important to use adequate monitoring tools to account for the concurrent dramatic increase in the life expectancy of PHWA (Polesel *et al*, 2010).

The SIRs for a few NADCs (anus, liver, non-melanomatous skin, and Hodgkin's lymphoma) increased in the SHCS after the introduction of HAART. At the same time, however, SHCS participants had grown older (the proportion of person-years from PWHA aged ⩾35 years had increased from 38% in the pre-, to 67% in the early-, and 81% in the late-HAART period).

The SIRs, however, are based on the indirect adjustment method, meaning that a standard population (the general Swiss population) provides the rates, and the study population (PWHA) provides the weights (age and sex structure) (Schoenbach, 2000). Hence, if the PWHA age structure changes substantially, the comparison of SIRs over time becomes problematic. Using, more correctly, IRs age-standardised by the direct method (the application of age-specific IRs among PWHA in different periods to the age and sex structure of the same standard population, i.e., PWHA over the entire study), cancers of the anus and non-melanomatous skin were the only malignancies to increase significantly, and only between the pre- and the early-HAART periods in the SHCS. Although the direct method gives greater comparability, it requires more data than the indirect method (Schoenbach, 2000). Therefore, even for the most frequent NADCs, the low number of cancer cases made the standardisation process unstable. Indeed, the 95% CI of age-specific IRs in PWHA in the pre-HAART and HAART periods broadly overlapped for individual NADCs, as well as for all NADCs combined.

Five individual NADCs showed a clear excess in the HAART periods among PWHA compared with the general population, regardless of statistical approach. Three of these NADCs (anus, liver, and Hodgkin's lymphoma) have a well-established viral aetiology (human papillomavirus (IARC, 2007), hepatitis B and C viruses (IARC, 1994), and Epstein–Barr virus (IARC, 1997), respectively). Contrary to KS and some types of NHL, a long latent period separates the infection with the responsible virus and the onset of cancer of the anus and liver. A viral aetiology for non-melanomatous skin cancer is not yet well established, except for the rare polyomavirus-associated Merkel cell carcinoma (Lanoy *et al*, 2009). Previous studies suggested that smoking could not totally account for the increases in lung cancer (The D:A:D Study Group, 2006; Chaturvedi *et al*, 2007), and that impaired immunity was associated with liver disease and liver cancer among PWHA infected with hepatitis B and C viruses (Clifford *et al*, 2008). The effect of HIV-related immunodeficiency on cancers of the lung and liver remains, however, ill-defined (Bouvard *et al*, 2009).

The SHCS has many strengths, including the length and completeness of follow-up and the good representation of women and different HIV-transmission categories (The Swiss HIV Cohort Study, 2010). Furthermore, the SHCS has been estimated to cover 45% of the cumulative number of HIV infections declared to the Swiss health authorities and 69% of registered AIDS cases, making the study unique in terms of population representativeness. The possibility of record linkage with high-quality cancer registries improved the completeness and quality of cancer-related information (e.g., histological confirmation) beyond what was available in the SHCS records.

The most important limitations of this study are 1) the relatively small number of individual NADCs, and 2) the complexity of adjusting for large decreases in competing non-cancer risks among PWHA (Keiser *et al*, 2004) without making implausible assumptions about the way removal of competing risks would have affected cancer risk (Rothman *et al*, 2008). Problems typical of record-linkage studies, for example, inaccuracies in personal identifiers and self-reported legal residence that may result in some undermatching between the SHCS participants and cancer registry records, cannot be completely excluded, but should have been largely avoided. With respect to surveillance bias, no difference was found in the risk of cancers in which screening can make a substantial difference (e.g., cancer of the colon–rectum, breast, prostate, and thyroid). Part of the excess of cancer of the anus and non-melanomatous skin, however, may be due to a more intense search and more accurate diagnosis of these cancers in PWHA than in the general population.

In conclusion, our study showed that the incidence of KS and NHL continued to decline from the early- to the late-HAART period, but the incidence of NADCs did not change substantially. Estimates of the excess of cancers of the anus, liver, lung, non-melanomatous skin and Hodgkin's lymphoma in PWHA compared with the general population (expressed as SIRs or age-specific IRs) became more precise in the HAART periods. Comparisons of the incidence of NADCs among PWHA in the pre-HAART and HAART periods remained very difficult, but they did not support claims of an adverse influence of HAART *per se* or HAART-related immune reconstitution on cancer risk.

## Figures and Tables

**Table 1:** Observed and expected numbers of cancers, SIRs, and 95% CIs among PWHA by period of cancer diagnosis

	**Period**								
	**Pre-HAART (1985–1996) 20 615 PY**			**Early-HAART (1997–2001) 17 690 PY**			**Late–HAART (2002–2006) 15 410 PY**		
**Cancer site or type (ICD10)**	**O/E**	**SIR**	**95% CI**	**O/E**	**SIR**	**95% CI**	**O/E**	**SIR**	**95% CI**
*AIDS-defining cancers*									
Kaposi's sarcoma (C46)	272/1.1	246	218–277	35/0.7	47.8	33.3–66.6	14/0.6	22.9	12.5–38.5
Non-Hodgkin's lymphoma (C82-C88, C96)	191/1.9	103	88.8–119	52/1.9	26.7	19.9–35.1	32/2.0	16.2	11.1–22.9
Cervix uteri (C53)	4/0.5	8.4	2.2–21.8	2/0.5	3.7	0.3–13.6	0/0.5	—	—
All AIDS-defining cancers	467/3.4	136	124–149	89/3.2	27.7	22.2–34.1	46/3.1	14.7	10.8–19.6
									
*Non-AIDS-defining cancers*									
Head and neck (C00-C14, C30-C32)^a^	9/2.1	4.3	2.0–8.3	8/2.7	2.9	1.3–5.8	7/3.2	2.2	0.9–4.5
Stomach (C16)	1/0.7	1.5	0.0–8.5	1/0.9	1.2	0.0–6.7	1/1.0	1.0	0.0–5.6
Small intestine, colon, rectum and rectosigmoid junction (C17-C20)	2/1.8	1.1	0.1–4.0	2/2.8	0.7	0.1–2.6	1/3.5	0.3	0.0–1.6
Anus (C21)	2/0.1	25.7	2.4–94.5	12/0.1	112	57.8–197	6/0.1	49.9	18.0–109
Liver (C22)	2/0.4	5.5	0.5–20.2	7/0.7	10.7	4.2–22.2	5/0.8	6.1	1.9–14.3
Pancreas (C25)	0/0.4	—	—	2/0.6	3.4	0.3–12.4	1/0.7	1.4	0.0–7.8
Trachea, lung and bronchus (C33, C34)	8/2.4	3.3	1.4–6.6	10/3.6	2.8	1.3–5.1	12/4.6	2.6	1.3–4.6
Skin, melanomatous (C43)	3/2.5	1.2	0.2–3.5	2/3.1	0.6	0.1–2.4	6/3.1	2.0	0.7–4.3
Skin, non-melanomatous (C44)	7/4.0	1.7	0.7–3.6	23/6.5	3.5	2.2–5.3	23/7.0	3.3	2.1–4.9
Breast (C50)^b^	1/1.7	0.6	0.0–3.4	4/3.3	1.2	0.3–3.2	4/4.3	0.9	0.2–2.4
Ovary (C56)	2/0.3	6.4	0.6–23.5	0/0.4	—	—	0/0.5	—	—
Prostate (C61)	0/1.1	—	—	5/2.8	1.8	0.6–4.1	5/3.8	1.3	0.4–3.1
Testis (C62)	4/3.0	1.3	0.3–3.4	3/2.4	1.2	0.2–3.7	1/1.8	0.5	0.0–3.1
Kidney (C64)	1/0.5	1.9	0.0–10.6	1/0.8	1.3	0.0–7.2	3/1.0	3.1	0.6–9.2
Bladder (C67)	1/1.0	1.1	0.0–6.0	0/1.4	—	—	1/1.7	0.6	0.0–3.3
Brain, meninges and central nervous systems (C70-C72)	2/0.9	2.2	0.2–7.9	2/0.9	2.2	0.2–8.0	0/1.0	—	—
Thyroid (C73)	2/0.7	2.9	0.3–10.7	1/0.8	1.2	0.0–7.0	0/0.8	—	—
Hodgkin's lymphoma (C81)	7/0.8	9.2	3.6–19.0	12/0.6	21.0	10.8–36.8	13/0.5	28.1	14.9–48.2
Multiple myeloma (C90)	3/0.2	14.8	2.8–43.9	1/0.3	3.2	0.0–18.1	2/0.4	5.1	0.5–18.9
Leukaemias (C91-C95)	1/0.7	1.5	0.0–8.3	2/0.8	2.4	0.2–8.8	1/0.9	1.1	0.0–6.5
All non-AIDS-defining cancers^c^	62/26.7	2.3	1.8–3.0	100/37.3	2.7	2.2–3.3	94/42.8	2.2	1.8–2.7
									
All cancers	529/30.1	17.6	16.1–19.1	189/40.6	4.7	4.0–5.4	140/46.0	3.0	2.6–3.6

Abbreviations: CI=confidence intervals; E=expected; HAART=highly active antiretroviral therapy; O=observed; PWHA=people with HIV or AIDS; PY=person years; SIRs=standardised incidence ratios.

a Includes nine tongue, six mouth, three larynx cancers, and one cancer each of lip, tonsil, oropharynx, nasopharynx, hypopharynx, and nasal cavity.

b Females only.

c Also includes four unknown primary sites plus one cancer each of the oesophagus, biliary tract, connective/soft tissue, and corpus uteri.

**Table 2:** IRs^a^ ( × 100 000), and IR ratios and corresponding 95% CIs for selected cancers^b^ among PWHA by period of cancer diagnosis

	**Period**										
	**Pre-HAART (1985–1996) 20 615 PY**			**Early-HAART (1997–2001) 17 690 PY**			**Late-HAART (2002–2006) 15 410 PY**			**IR ratio (95% CI)**	**IR ratio (95% CI)**
**Cancer site or type (ICD10)**	**O**	**IR**	**95% CI**	**O**	**IR**	**95% CI**	**O**	**IR**	**95% CI**	**early *vs* pre**	**late *vs* early**
*AIDS-defining cancers*											
Kaposi's sarcoma (C46)	272	1375	1213–1551	35	194	123–285	14	66.9	29.5–123	0.1 (0.1–0.2)	0.3 (0.2–0.7)
Non-Hodgkin's lymphoma (C82-C88, C96)	191	952	818–1102	52	252	176–347	32	98.4	66.1–141	0.3 (0.2–0.4)	0.4 (0.2–0.6)
All AIDS-defining cancers^c^	467	2283	2075–2505	89	442	336–566	46	163	112–226	0.2 (0.1–0.3)	0.4 (0.2–0.6)
											
*Non-AIDS-defining cancers*											
Head and neck (C00-C14, C30-C32)	9	41.6	18.8–79.3	8	37.2	15.0–75.3	7	19.2	7.7–39.8	0.9 (0.3–2.4)	0.5 (0.2–1.4)
Anus (C21)	2	8.3	0.9–30.1	12	42.7	21.9–74.7	6	25.3	7.3–58.8	5.2 (1.3–21.3)	0.6 (0.2–1.7)
Liver (C22)	2	9.6	1.1–34.7	7	25.9	10.4–53.5	5	17.1	5.1–40.9	2.7 (0.5–13.9)	0.7 (0.2–2.1)
Trachea, lung, and bronchus (C33, C34)	8	38.1	16.3–75.3	10	36.5	17.4–67.2	12	33.9	17.2–59.7	1.0 (0.4–2.4)	0.9 (0.4–2.2)
Skin, non-melanomatous (C44)	7	35.8	14.1–74.3	23	85.7	53.7–129	23	86.8	48.1–140	2.4 (1.0–5.7)	1.0 (0.5–1.9)
Hodgkin's lymphoma (C81)	7	30.7	12.2–63.5	12	42.9	20.8–77.0	13	52.8	20.2–102	1.4 (0.5–3.7)	1.2 (0.5–3.1)
All non-AIDS-defining cancers	62	320	242–414	100	397	316–491	94	324	250–410	1.2 (0.9–1.7)	0.8 (0.6–1.1)
											
All cancers	529	2602	2379–2840	189	839	704–990	140	487	395–590	0.3 (0.3–0.4)	0.6 (0.4–0.8)

Abbreviations: CI=confidence interval; HAART=highly active antiretroviral therapy; IR=incidence rate; O=observed; PWHA=people with HIV or AIDS; PY=person years.

a Rates are standardised by 5-year age group and sex, based on the age and sex distribution of all PWHA included in this study;

b Cancers diagnosed in >10 PWHA in the HAART period;

c Including cervix uteri (C53).

## Appendix

The members of the Swiss HIV Cohort Study are M Battegay, E Bernasconi, J Böni, HC Bucher, P Bürgisser, A Calmy, M Cavassini, R Dubs, M Egger, L Elzi, M Fischer, M Flepp, A Fontana, P Francioli (President of the SHCS), H Furrer (Chairman of the Clinical and Laboratory Committee), CA Fux, M Gorgievski, HF Günthard (Chairman of the Scientific Board), HH Hirsch, B Hirschel, I Hösli, C Kahlert, L Kaiser, U Karrer, C Kind, T Klimkait, B Ledergerber, G Martinetti, B Martinez de Tejada, N Müller, D Nadal, F Paccaud, G Pantaleo, A Rauch, S Regenass, M Rickenbach (Head of Data Center), C Rudin (Chairman of the Mother and Child Substudy), P Schmid, D Schultze, F Schöni-Affolter, J Schüpbach, R Speck, P Taffé, A Telenti, A Trkola, P Vernazza, R Weber, and S Yerly.
